# Contribution of Schwann Cells to Remyelination in a Naturally Occurring Canine Model of CNS Neuroinflammation

**DOI:** 10.1371/journal.pone.0133916

**Published:** 2015-07-21

**Authors:** Kristel Kegler, Ingo Spitzbarth, Ilka Imbschweiler, Konstantin Wewetzer, Wolfgang Baumgärtner, Frauke Seehusen

**Affiliations:** 1 Department of Pathology, University of Veterinary Medicine, Hannover, Germany; 2 Center of Systems Neuroscience, Hannover, Germany; 3 Department of Functional and Applied Anatomy, Center of Anatomy, Hannover Medical School, Hannover, Germany; Hospital Nacional de Parapléjicos - SESCAM, SPAIN

## Abstract

Gliogenesis under pathophysiological conditions is of particular clinical relevance since it may provide evidence for regeneration promoting cells recruitable for therapeutic purposes. There is evidence that neurotrophin receptor p75 (p75^NTR^)-expressing cells emerge in the lesioned CNS. However, the phenotype and identity of these cells, and signals triggering their *in situ* generation under normal conditions and certain pathological situations has remained enigmatic. In the present study, we used a spontaneous, idiopathic and inflammatory CNS condition in dogs with prominent lympho-histiocytic infiltration as a model to study the phenotype of Schwann cells and their relation to Schwann cell remyelination within the CNS. Furthermore, the phenotype of p75^NTR^-expressing cells within the injured CNS was compared to their counter-part in control sciatic nerve and after peripheral nerve injury. In addition, organotypic slice cultures were used to further elucidate the origin of p75^NTR^-positive cells. In cerebral and cerebellar white and grey matter lesions as well as in the brain stem, p75^NTR^-positive cells co-expressed the transcription factor Sox2, but not GAP-43, GFAP, Egr2/Krox20, periaxin and PDGFR-α. Interestingly, and contrary to the findings in control sciatic nerves, p75^NTR^-expressing cells only co-localized with Sox2 in degenerative neuropathy, thus suggesting that such cells might represent dedifferentiated Schwann cells both in the injured CNS and PNS. Moreover, effective Schwann cell remyelination represented by periaxin- and P0-positive mature myelinating Schwann cells, was strikingly associated with the presence of p75^NTR^/Sox2-expressing Schwann cells. Intriguingly, the emergence of dedifferentiated Schwann cells was not affected by astrocytes, and a macrophage-dominated inflammatory response provided an adequate environment for Schwann cells plasticity within the injured CNS. Furthermore, axonal damage was reduced in brain stem areas with p75^NTR^/Sox2-positive cells. This study provides novel insights into the involvement of Schwann cells in CNS remyelination under natural occurring CNS inflammation. Targeting p75^NTR^/Sox2-expressing Schwann cells to enhance their differentiation into competent remyelinating cells appears to be a promising therapeutic approach for inflammatory/demyelinating CNS diseases.

## Introduction

Following injury, the peripheral nervous system (PNS) possesses a pronounced regenerative capacity, while regeneration is insufficient and remains abortive in central nervous system (CNS) diseases [1, 2]. The relatively enhanced regeneration of the PNS is in part attributed to the plasticity of Schwann cells, the major class of PNS glia [3, 4, 5]. Schwann cells undergo a remarkable transformation in response to injury, characterized by a transient period of proliferation and extensive changes in gene expression [6]. Although many of these molecular changes result in a cellular status reminiscent of immature Schwann cells [3, 5], recent work implies that the post-injury stage of Schwann cells represents an unique phenotype, promoting repair and lacking several features found in other differentiation stages of the Schwann cell lineage [7].

Although Schwann cells are not a physiological component of the CNS, recent evidence indicates that they crucially contribute to the cellular response following CNS injury under certain circumstances. Schwann cell participation has been largely described in experimental animal models for spinal cord trauma and toxic demyelination caused by injection of substances such as kainate, ethidium bromide, 6-aminonicotinamide, and lysolecithin [8, 9, 10, 11]. Interestingly, Schwann cell-mediated remyelination is a well-known phenomenon in the spinal cord of patients suffering from multiple sclerosis (MS), the major human demyelinating condition [12, 13, 14, 15, 16]. Although data upon the exact role of these cells in terms of functional effects are lacking so far, it is suggested that Schwann cells might contribute to significant CNS regeneration. Their origin, however, in naturally occurring diseases remains unclear so far. In particular, it remains to be determined whether the presence of an immature or post-injury Schwann cell phenotype promotes CNS regeneration under natural circumstances.

Strikingly, the origin of Schwann cells within the CNS is controversially discussed [10, 11, 17, 18]. On the one hand, experimental and naturally occurring spinal cord injury studies demonstrated that immature/dedifferentiated Schwann cells expressing the prototype marker p75 neurotrophin receptor (p75^NTR^) migrate into the lesioned site from PNS sources such as spinal nerve roots [12, 19, 20, 21]. On the other hand, lineage-tracing studies have clearly shown that CNS-resident precursors are the major source of Schwann cell-mediated remyelination within toxic CNS demyelination lesions of mice, while only very few remyelinating Schwann cells invade the CNS from PNS sources [11]. Additionally, *in vitro* studies suggest that p75^NTR^-expressing Schwann cells derived from the CNS share several properties with oligodendrocyte precursor cells (OPCs), including similar voltage-gated potassium channels (K_v_) activation and antigenic expression, substantiating that these cells might represent centrally generated, pre-myelinating Schwann cells [22, 23, 24] However, the relationship between canine CNS Schwann cells and OPCs *in vivo* remained unresolved. Irrespective of their exact origin, it remains to be resolved, which mechanisms function as triggering factors for the occurrence of Schwann cells in the CNS.

To address the former aspects, we aimed to investigate naturally occurring lympho-histiocytic encephalitis and granulomatous meningoencephalitis (GME), two CNS idiopathic inflammatory entities of dogs, grouped as non-suppurative meningoencephalitis of unknown origin [25]. The suitability of this model is based on several observations. First, it belongs to a group of idiopathic diseases with suspected autoimmune and/or multifactorial etiopathogenesis with no infectious etiology proved to date [26, 27], thus resembling the etiopathogenesis of multiple sclerosis (MS) or other immune-mediated CNS diseases in some aspects [28, 29]. Secondly, similar to MS [29], etiologically undertermined lympho-histiocytic encephalitis and GME are characterized by a multifocal distribution pattern, involving primarily the white matter and perivascular infiltration consisting mainly of macrophages and T cells [26, 30, 31]. Thirdly, mRNA expression and protein levels of IL-17 and IFN-γ detected in lesions of dogs suffering from GME are comparable to those described in MS and murine experimental autoimmune encephalitis (EAE) [32]. In addition, mRNA expression of chemokine receptors, such as chemokine (C-X-C motif) receptor 3 (CXCR3), C-C chemokine receptor type 2 (CCR2), and C-C chemokine receptor type 4 (CCR4), also implicated in MS and EAE pathogenesis [33], are described to play a role in the formation of GME lesions [32]. Finally, many conditions in the species dog share striking similarities with their human counterparts thus representing suitable translational models for studying human aging [34, 35], spinal cord injury [36] and MS, as described in the canine distemper virus (CDV)-induced demyelination model [22, 37]. Moreover, canine glial cells recently gained attention in cell-based therapies [38, 39], which is in part due to their primate-like properties regarding *in vitro* proliferation and marker expression [40, 41, 42]. The major strength of the model is based on the spontaneous occurrence of the lesions. Thus, the species dog might help to overcome the gap between highly homogenous and standardized lesions in experimental rodent models and clinically relevant conditions in humans.

In this study, we analyzed the spatial distribution and the identity of p75^NTR^-expressing cells and mature myelinating Schwann cells within the brains of dogs with non-suppurative meningoencephalitis of unknown origin, and compared the observed expression profiles with those of a case of peripheral nerve injury, and with healthy sciatic nerves and canine brains. This is the first study, which characterizes in detail the Schwann cell phenotype in the injured brain, therewith providing novel insights into the involvement of Schwann cells under naturally occurring pathophysiological circumstances in the CNS.

## Materials and Methods

### Ethics statement

All formalin fixed and paraffin embedded archived material used in this study was collected by one of the authors (WB) during his work at the diagnostic pathology services of the Department of Pathology, University of Veterinary Medicine Hannover. The majority of the brain samples have been used in a previous study [25]. The present study was conducted in accordance with the German Animal Welfare Act. The authors confirm that no animals were infected or sacrificed for the purpose of this retrospective pathological study. This study is not an animal experiment since all animals were dead at the time of submission for necropsy in order to investigate the causes of death and disease. In cases in which euthanasia was performed because of poor prognosis, this procedure was done in the respective Veterinary Hospital before the patient was submitted to the diagnostic service of the Department of Pathology. All dog owners provided written consent for the dogs’ tissues to be collected and used for research purposes.

For the *in vitro* investigations, post mortem brain tissue of four beagle dogs was used. The dogs served as a control animal in an unrelated animal experiment, which was conducted in compliance with the law of animal welfare, Germany (permission numbers: 33.9-42502-05-13A346).

### Animals, histology and neuropathological diagnoses

Following routine necropsy, brain samples of dogs suffering from spontaneous CNS diseases were collected and subsequently fixed in non-buffered formalin (10%) and embedded in paraffin. Serial sections (3 μm thick) were mounted on SuperFrost-Plus slides (Menzel Gläser, Braunschweig, Germany), and stained with hematoxylin and eosin (HE) for neuropathological classification. Neuropathological diagnosis was done by board certified veterinary pathologists on HE sections and classified as non-suppurative meningoencephalitis of unknown etiology comprising granulomatous and lympho-histiocytic meningoencephalitis (n = 25). All cases were examined immunohistochemically for 18 different infectious agents, including viruses, bacteria and prion protein [25], and only those cases with undetermined etiology were included in this study. In addition, formalin-fixed and paraffin-embedded samples of a sciatic nerve of a dog suffering from degenerative neuropathy of unknown origin, characterized by multifocal spheroid formation, dilatation of myelin sheaths and myelinophagia was included to compare the Schwann cell phenotypes in injured peripheral nerve and brain. Furthermore, archived formalin-fixed and paraffin-embedded brain tissue of five dogs lacking morphological evidence of CNS disease, and two healthy sciatic nerves were selected from the archives of the Department of Pathology and were used as controls. Sex, age, and morphologic diagnoses from diseased dogs as well as control dogs are summarized in Table 1.

**Table 1:** Sex, age, anatomic localization of the lesion, morphologic diagnosis, distribution of the lesion, severity of the lesion and expression of p75^NTR^ in dogs suffering non-suppurative meningoencephalitis of unknown origin, degenerative neuropathy and control dogs.

Dog	Sex	Age	Anatomic localization of the lesion	Diagnosis	Distribution of the lesion	Severity	p75^NTR^ expression
1	M	4 yrs	cerebrum	GME	focal extensive	severe	+
2	M	3 yrs	cerebellum, brain stem	GME	multifocal	severe	-
3	M	6 yrs	cerebellum, brain stem	GME	multifocal	moderate	-
4	F	3 yrs	cerebrum,	GME	focal extensive	severe	+
5	M	12 yrs	cerebellum, brain stem	GME	multifocal	severe	-
6	F	7 yrs	cerebrum	GME	focal extensive	severe	+
7	M	1 yr	cerebellum, brain stem	GME	multifocal	severe	+
8	F	7 yrs	cerebellum, brain stem	GME	multifocal	severe	+
9	M	10 yrs	cerebrum	GME	multifocal	moderate	+
10	M	7 yrs	cerebrum	GME	multifocal	severe	-
11	M	8 yrs	cerebellum, brain stem	GME	multifocal	moderate	+
12	M	4,5 yrs	cerebrum	GME	multifocal	mild	-
13	M	4 mo	cerebellum	GME	multifocal	moderate	-
14	F	4 yrs	cerebrum	GME	multifocal	mild	-
15	F	4 yrs	cerebellum, brain stem	GME	multifocal	moderate	+
16	M	9 yrs	cerebrum	GME	multifocal	moderate	+
17	F	4,5 yrs	cerebrum	GME	multifocal	moderate	+
18	M	5 yrs	cerebrum	GME	focal extensive	severe	+
19	F	8 yrs	cerebrum	GME	multifocal	severe	+
20	F	5 yrs	cerebellum, brain stem	Lympho-histiocytic ME	multifocal	moderate	+
21	F	8,5 ysr	cerebrum	Lympho-histiocytic ME	multifocal	mild	+
22	M	3 mo	cerebrum	Lympho-histiocytic ME	multifocal	mild	-
23	F	4,5 yrs	cerebellum, brain stem	Lympho-histiocytic ME	multifocal	mild	-
24	M	1 yr	cerebrum	Lympho-histiocytic ME	multifocal	moderate	-
25	M	2,5 yrs	cerebrum	Lympho-histiocytic ME	multifocal	mild	-
26	M	7 yrs	sciatic nerve	Degenerative neuropathy	diffuse	moderate	+
27	M	6 mo	cerebrum, cerebellum, brains stem, sciatic nerve	Healthy (control dog)	n.a	n.a	-
28	M	6 mo	cerebrum, cerebellum, brains stem	Healthy (control dog)	n.a	n.a	-
29	F	12 yrs	cerebrum, cerebellum, brains stem	Chronic hepatitis (control dog)	n.a	n.a	-
30	F	4 weeks	cerebrum, cerebellum, brains stem, sciatic nerve	Megaesophagus (control dog)	n.a	n.a	-
31	F	9 yrs	cerebrum, cerebellum, brains stem	Anaplastic carcinoma of the mamary gland (control dog)	n.a	n.a	-

M: male; F: female; GME: granulomatous meningoencephalitis; ME: meningoencephalitis; yrs: years; mo: months; n.a: not applicable; (+): positive; (-): negative.

In non-suppurative meningoencephalitis, lesioned areas were defined by the presence of the characteristic perivascular inflammatory infiltrates composed of macrophages, lymphocytes and scattered plasma cells [26, 30, 31]. Well-demarcated, single perivascular cuffing with inflammatory cells infiltrating the parenchyma, or confluent perivascular infiltrates with no clear separation between affected blood vessels were considered each as one lesioned area. A total of 198 lesioned areas comprising all 25 dogs suffering from non-suppurative meningoencephalitis were evaluated. Additionally, to assess the spatial distribution of p75^NTR^-expressing cells in diseased dogs, lesioned areas localized within the grey and white matter of the cerebrum (n = 108; grey matter: n = 34; white matter: n = 74), cerebellum (n = 52; grey matter: n = 5; white matter: n = 47), and the brain stem (n = 38) were separately analyzed.

### Antibodies and lectins

For immunohistochemistry (IHC) and immunofluorescence (IF) monoclonal antibodies included antibodies against the neurotrophin receptor p75 (p75^NTR^, clone HB8737, 1:5 IHC, 1:2 IF, ATCC, Rockville, USA), phosphorylated neurofilaments (p-NF, SMI 312, 1:1,000 IHC, Sternberger Monoclonals, Lutherville, USA), β-amyloid precursor protein (β-APP, MAB348; 1:800 IHC, Chemicon International, Temecula, USA), CD3 (A0452, 1:300 IHC, DakoCytomation, Hamburg, Germany), and the major peripheral myelin protein zero (P0; clone P07, 1:1,000 IF, [43]). Polyclonal antibodies included anti-glial fibrillary acidic protein (GFAP, Z0334, 1:1,000 IHC, 1:400 IF, DakoCytomation, Hamburg, Germany), anti-early growth response 2 (Egr2/Krox20, LS-B3577, 1:500 IHC, 1:200 IF, LifeSpan BioSciences, Inc., Seattle, USA), anti-growth associated protein-43 (GAP-43, AB5220, 1:600 IHC, 1:200 IF, Millipore, Darmstadt, Germany), anti-periaxin (PRX, HPA001868, 1:5000 IHC, 1:1000 IF, Sigma-Aldrich, St. Louis, USA), anti-PDGFR-α (C-20:sc-338, 1:400 IHC, 1:200 IF, Santa Cruz Biotechnology, Inc., Dallas, USA), and the anti-sex determining region Y-box 2 (Sox2, D6D9, 1:50 IHC, 1:20 IF, Cell Signaling Technology, Inc., Danvers, USA). The lectin *Bandeiraea simplicifolia* was used to detect microglia/macrophages (BS-1, L3759, Sigma-Aldrich, Taufkirchen, Germany).

### Immuno- and lectin histochemistry

Immuno- and lectin histochemistry was performed by using the avidin-biotin-peroxidase complex (ABC) method as previously described [36, 44]. Briefly, 3 μm thick sections were deparaffinized and rehydrated through a graded series of alcohols, treated with 0.5% H_2_O_2_ to block endogenous peroxidase and, heated in sodium-citrate buffer for 30 minutes in the microwave for antigen retrieval. After incubation with 20% goat serum, sections were incubated with the respective primary antibody (Ab) overnight at 4°C. As negative control, primary antibodies were substituted with either rabbit serum for polyclonal Abs (1:3000; R4505; Sigma Aldrich, Taufkirchen, Germany) or mouse Balb/c serum for monoclonal Abs (1:1000; CBL600; Millipore, Schwalbach, Germany). Biotinylated goat-anti-rabbit IgG (BA-1000) or goat-anti-mouse IgG (BA-9200), diluted 1:200 (Vector Laboratories, Burlingame, CA, USA), were used as secondary antibodies. Color development was done using 3,3’-diaminobenzidine tetrahydrochloride (DAB) with H_2_O_2_ (0.03%, pH 7.2) for 5 min followed by slight counterstaining with Mayer’s hemalaun. For double immunostaining, p-NF was combined with periaxin. Immunostaining for both antigens was done sequentially. Visualization of the second antigen was done by using HistoGreen as a substrate (E109, Linaris, Wertheim-Bettingen, Germany).

### Double immunofluorescence staining

On representative sections, immunofluorescence double staining procedures were performed as described [44] on 3 μm thick paraffin-embedded sections to demonstrate a possible co-localization of p75^NTR^ with PDGFR-α, the transcription factors Sox2 and Egr2/Krox20, GAP-43, GFAP, and periaxin. In addition, double labeling for P0 and periaxin was performed. Briefly, sections were simultaneously incubated with the respective primary antibodies for 90 min. Cy3-labeled goat anti-mouse (red, 1:200, Alexa Fluor 555 dye, Life Technologies) and Cy2-labeled goat anti-rabbit (green, 1:200, Alexa Fluor 488 dye, Life Technologies) secondary antibodies were used to visualize the respective antigens. Nuclear counterstaining was performed with 0.01% bisbenzimide (H33258, Sigma Aldrich, Taufkirchen, Germany) and sections were mounted with Dako Fluorescent Mounting medium (DakoCytomation, Hamburg, Germany).

### Organotypic slice cultures of the adult canine brain stem

Organotypic slice cultures from four healthy dogs were established according to Heimrich and Frotscher, 1993 [45] with modifications [22, 40, 46]. Briefly, the isolated brain stems were placed on a plate of low-adhesive plastic (Vulcolan) and sections were cut (350 μm) using a tissue chopper (Mc Illwain, Mickle Lab, Engineering Surrey, UK). Slices were transferred to organotypic inserts (Millicell Cell Culture Inserts, Millipore, Schwalbach, Germany), placed in 6-well plates (Nunclon, Nunc, Wiesbaden, Germany), and maintained in Dulbecco’s modified Eagle (DME) medium (Invitrogen Inc., Karlsruhe, Germany) containing 10% fetal calf serum (PAA, Marburg, Germany). For each time point, slices were cut out of the membrane using a scalpel and fixed in paraformaldehyde (4% in PBS). After paraffin embedding, sections (4 μm) were cut and mounted on slides, as described above. The slices were evaluated at days 0, 3, 9, and 18 in culture. Inmmunostaining for BS-1 and periaxin, and double immunofluorescence procedures with anti-p75^NTR^ and anti-Sox2 antibodies were done as described above.

### Statistical analysis

The obtained immuno- and lectin histochemical signals were evaluated quantitatively by counting the number of positive structures (cells and axons), using a morphometric grid. All lesioned areas within each case were evaluated. According to the individual size of the respective area, the total lesioned area or, in cases of large lesions, a maximum of 12 randomly distributed fields were counted. The number of immunoreactive structures per mm² was calculated and used for statistical analyses. Immunohistochemical data were subjected to statistical analysis using “SPSS” software for Windows (SPSS Inc., Chicago, IL, USA), version 21.0. As part of the data was not normally distributed, the Kruskal-Wallis test and subsequent Mann-Whitney U-tests for group-wise comparisons were employed, and p values lower than 0.05 were considered statistically significant. All box-plots show median value, quartiles, minimum and maximum of positive signals. In order to reveal potential co-dependencies between the different immuno- and lectin-histcohemical markers, correlation coefficients were calculated using the Spearman’s rank correlation test. For double immunofluorescence in non-supurative meningoencephalitis, the total number of p75^NTR^-expressing cells and the number of co-localization with the respective antibodies per lesioned area were counted and are expressed in percentage of total cells. In the case of degenerative neuropathy and healthy control sciatic nerves, a maximum of 10 areas was randomly selected and the number of cells with co-localization of the respective antibodies was counted. The results are expressed in percentage of total cells counted.

## Results

### Distribution of p75^NTR^-expressing cells in canine non-suppurative meningoencephalitis of unknown etiology

In all five control dogs, p75^NTR^ was limited to the neuropil of the trigeminal nucleus and tract, and in association with the leptomeningeal blood vessels and choroid plexus capillaries. In non-suppurative meningoencephalitis, glial expression of p75^NTR^ was found in 14 (56%) out of 25 dogs analyzed (Table 1). As the data suggested that there was no obvious predisposition concerning sex, age, and severity of lesions in the appearance of p75^NTR^-expressing cells within the injured brains, individual lesioned areas (n = 198) were used as individual objects for further statistical investigations. In lesioned areas localized in the cerebrum (n = 108), p75^NTR^-expressing cells were observed in 6 out of 34 (18%) areas distributed in the grey matter, and 26 out of 74 (35%) areas of the white matter. Within the cerebellum (n = 52), there were no p75^NTR^-expressing cells in the grey matter (n = 5), but they were detected in 27 out of 47 (57%) lesioned areas localized in the white matter. The brain stem displayed the highest percentage of lesioned areas with p75^NTR^-positive cells (24 out of 38; 63%).

In all areas, in which p75^NTR^-expressing glial cells were observed, the cells were exclusively present intralesionally, and displayed a spindle-shaped and bipolar morphology with an oval cell body and nucleus, reminiscent of Schwann cells [21, 22]. In addition, a small number of p75^NTR^-positive cells were characterized by multiple processes and a stellate-shaped morphology. All p75^NTR^-expressing cells were found in immediate vicinity to perivascular inflammatory infiltrates. Interestingly, p75^NTR^-immunoreactive cells were occasionally detected inside the Virchow-Robin spaces, intermingled with inflammatory cells. In addition, p75^NTR^-expressing cells were found in close proximity to the entry of cranial nerves in the brain stem. The facts that nearly all of the p75^NTR^-expressing cells were found in close vicinity to a possible entry point for PNS-resident Schwann cells, and that they clearly resemble normal Schwann cells morphologically might suggested that this CNS population of p75^NTR^-expressing cells is most probably constituted by invading Schwann cells originating from the PNS.

No immunoreactivity was present within the adjacent normally appearing neuroparenchyma. The emergence of p75^NTR^-expressing cells thus seems to represent a general phenomenon that takes place in multiple localizations, *i*.*e*. the injured cerebral white and grey matter, cerebellar white matter, and the brain stem.

### Phenotypical characterization of p75^NTR^-expressing cells reveals similarities with dedifferentiated Schwann cells in canine non-suppurative meningoencephalitis

p75^NTR^ represents the prototype marker for immature Schwann cells, the adult non-myelinating form, and dedifferentiated Schwann cells after peripheral nerve injury [3, 4, 5, 47], but is not able to discriminate between these differentiation stages. As the three Schwann cell phenotypes have been shown to be similar, but not antigenically identic [5, 7], we examined the expression of several markers in order to identify the phenotype of p75^NTR^-expressing cells within CNS lesion sites, in comparison with a case of degenerative neuropathy of the sciatic nerve and healthy sciatic nerves. Expression of GFAP and the transcription factor Sox2 is shared by immature, non-myelinating, and dedifferentiated Schwann cells in the PNS [48, 49, 50, 51]. GAP-43 expression, however, is mainly described in immature Schwann cells and the adult non-myelinating form [5, 52]. An antibody against Egr2/Krox20 was used to detect pro-myelinating and mature myelinating Schwann cells, as these differentiation stages have shown to express this transcription factor [51, 53]. Antibodies directed against periaxin and myelin protein zero (P0) were used in order to detect the mature myelinating phenotype of Schwann cells [54, 55].

**Fig 1:**
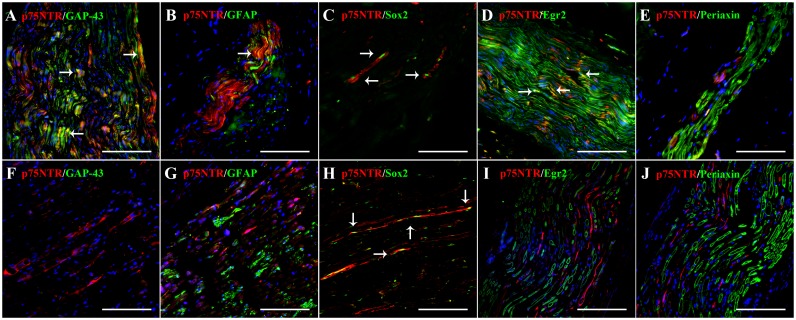
Double immunofluorescence staining in a control sciatic nerve of a 4 week-old dog (A-E) and sciatic nerve of a dog with degenerative neuropathy (F-J). In the control sciatic nerve, there is co-localization of p75^NTR^ (red) with GAP-43 (green, arrows, A), GFAP (green, arrows, B), and nuclear Sox2 (green, arrows, C), thus most probably representing immature and/or non-myelinating Schwann cells. Co-expression of p75^NTR^ (red) and Egr2/Krox20 (green, arrows, D) suggests a transition state between an immature and a myelinating Schwann cell phenotype. Periaxin expression (green, E) does not co-localize with p75^NTR^ (red) in any cell, substantiating that p75^NTR^ is completely down-regulated, once Schwann cells begin to myelinate. In contrast to the healthy nerve, there is no co-expression of p75^NTR^ (red) with GAP-43 (green, F) and GFAP (green, G) in the case of degenerative neuropathy. However, co-localization of p75^NTR^ with nuclear Sox2 (green, arrows, H) is evident, suggesting a dedifferentiated Schwann cell phenotype. Egr2/Krox20 (green, I) and periaxin (green, J) do not show co-labeling with p75^NTR^ (red) in any cell. Nuclear counterstaining (blue) with bisbenzimide. (A), (B), (D), (F), (G), (I) and (J) scale bars: 100 μm. (C), (E) and (H) scale bars: 50 μm.

In control sciatic nerves, 8% of the total cells counted showed co-localization for p75^NTR^ and GAP-43 in the 4 week-old puppy, and 1% co-expressed both markers in the 6 month-old dog (Fig 1A). GFAP and p75^NTR^ co-localization was detected in 8% and 7% of the total cells counted in the 4 weeks-old and 6 month-old dogs, respectively (Fig 1B). Approximately 4% and 6% of the total cells counted were immunoreactive for both p75^NTR^ and Sox2 (Fig 1C), in the 4 weeks-old and 6 month-old dogs, respectively. Co-expression of p75^NTR^ and Egr2/Krox20 (Fig 1D) was observed in 7% of the total cells counted in the 4 week-old puppy, thus probably representing a pre-myelinating state. In sciatic nerves of both control dogs, periaxin did not co-localize with p75^NTR^ (Fig 1E) in any cell substantiating that p75^NTR^ was completely down-regulated once Schwann cells myelinate. Interestingly, in the case of degenerative neuropathy, p75^NTR^-expressing Schwann cells co-localized with Sox2 in 12% of the total cells counted, but not with any of the other markers tested (Fig 1F, 1G, 1H, 1I and 1J), thus suggesting that they represent a dedifferentiated Schwann cell phenotype.

**Fig 2:**
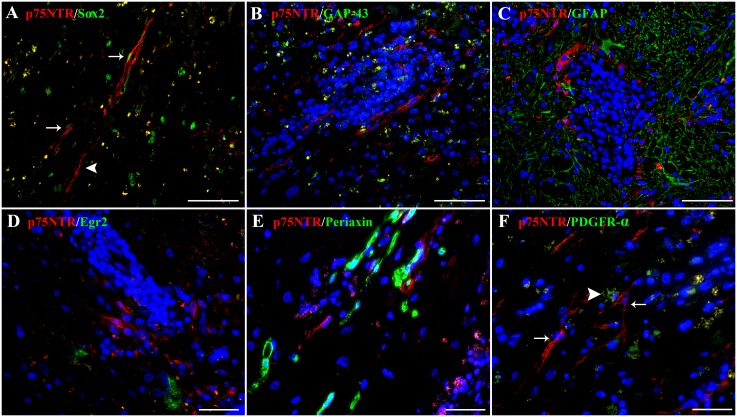
Double immunofluorescence staining in a representative case of canine non-suppurative meningoencephalitis of unknown etiology (dog number 18). Co-expression of p75^NTR^ (red, A) and Sox2 (green, A) is observed in bipolar cells (arrow), morphologically resembling Schwann cells, which are localized in close proximity to a blood vessel. Note that few p75^NTR^-expressing cells lack Sox2 expression (arrowhead). In contrast to Sox2, GAP-43 (green, B), GFAP (green, C), Egr2/Krox20 (green, D), periaxin (green, E), and PDGFR-α (green, arrowhead, F) are not co-expressed with p75^NTR^ (red), thus implying that p75^NTR^/Sox2-positive cells might represent a dedifferentiated Schwann cell phenotype within the injured CNS. Nuclear counterstaining (blue) with bisbenzimide. (A), (B) and (C) scale bars: 100 μm. (D), (E) and (F) scale bars: 50 μm.

In non-suppurative meningoencephalitis of unknown etiology, 63% of the p75^NTR^-positive cells, both with bipolar and multipolar morphology, co-expressed Sox2 (Fig 2A). Interestingly, GAP-43 (Fig 2B), GFAP (Fig 2C), Egr2/Krox20 (Fig 2D) and periaxin (Fig 2E) did not overlap with p75^NTR^ expression in any cell of the lesioned areas. Based on this observation, we sought to determine, whether the detected p75^NTR^ positive cells in fact may represent OPCs, since previous reports demonstrated that OPCs may express both p75^NTR^ [56, 57, 58] and Sox2 [59]. However, in all CNS lesioned areas examined, none of the p75^NTR^-expressing cells was found to co-localize with PDGFR-α (Fig 2F), the prototype marker for OPCs. These findings highly suggest that p75^NTR^-Sox2-positive cells within the CNS mainly represent dedifferentiated Schwann cells emerging after injury. Moreover, they appear to be comparable to a similar Schwann phenotype, which occurs in response to canine peripheral nerve disease.

### The occurrence of p75^NTR^-expressing cells is associated with effective Schwann cell mediated remyelination in canine non-suppurative meningoencephalitis

**Fig 3:**
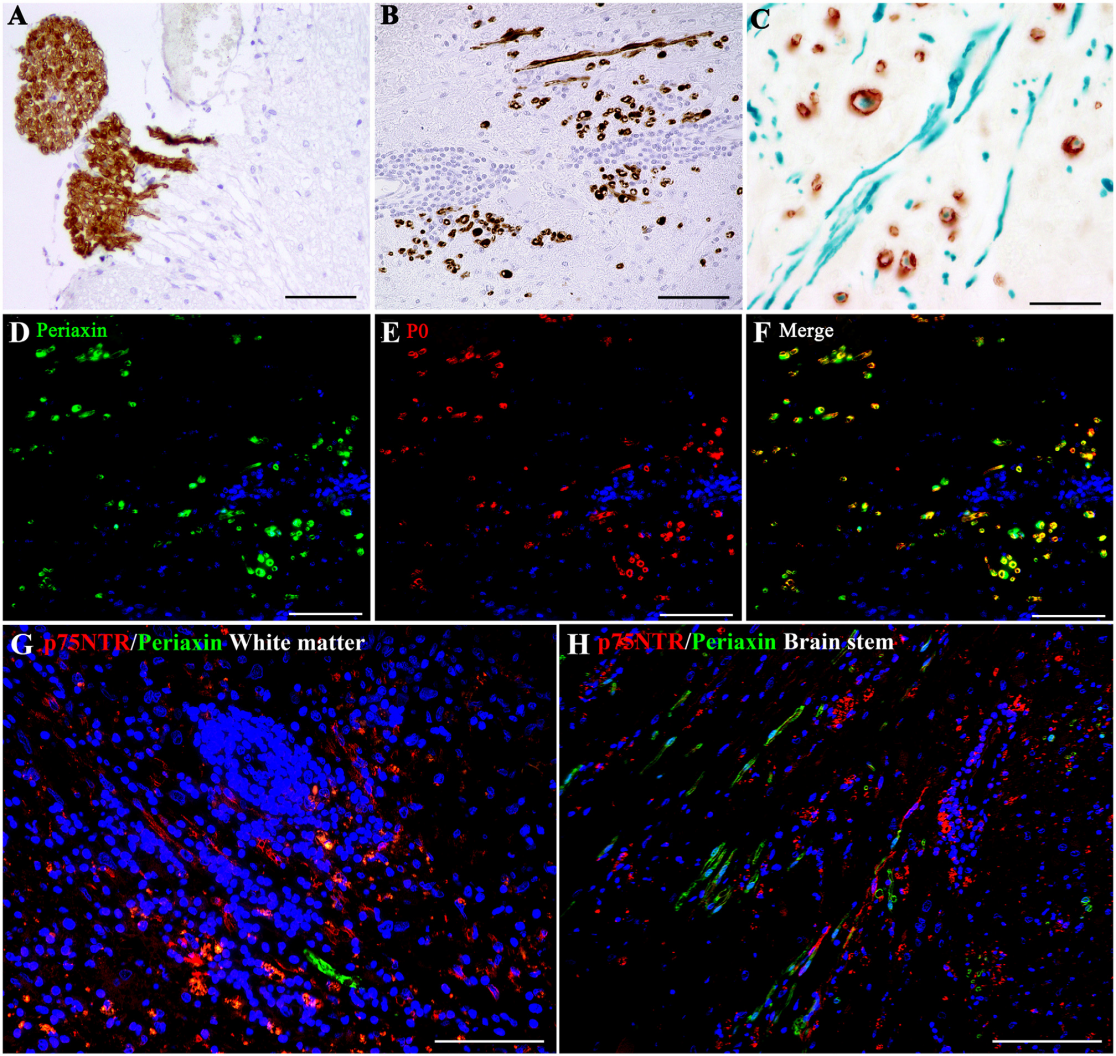
The immunoreaction of the peripheral myelin protein periaxin (brown, A) is limited to cranial nerves and abruptly stops upon entry of axons into the CNS in the brain stem of a control dog (dog number 27). In non-suppurative meningoencephalitis of unknown origin (dog number 18, B), intralesional periaxin-positive structures (brown) appear as round to oval, small, ring-like foci with occasional clear central cores, most probably representing myelinating Schwann cells ensheathing an axon. Double immunohistochemistry for periaxin (brown, C) and p-NF (phosphorylated neurofilament, green, C) verifies that a large proportion of periaxin-positive Schwann cells (brown) in fact enwrap axons (green), substantiating effective Schwann cell remyelination. (A) scale bar: 50 μm. (B) and (C) scale bars: 100 μm. Double immunofluorescence staining for periaxin (green, D) and P0 (red, E) in a representative case of canine non-suppurative meningoencephalitis of unknown etiology (dog number 18) reveals that virtually all periaxin-positive Schwann cells co-express P0 (F). Nuclear counterstaining (blue) with bisbenzimide. (D), (E) and (F) scale bars: 100 μm. Double immunofluorescence staining for p75^NTR^ (red) and periaxin (green) in the white matter (dog number 18; G) and brain stem (dog number 20; H) of representative cases of canine non-suppurative meningoencephalitis demonstrates increased Schwann cell remyelination depicted by periaxin immunoreactivity (green) in the brain stem compared to the cerebral white matter. Note that there is no co-localization of both markers. Nuclear counterstaining (blue) with bisbenzimide. (G) and (H) scale bars: 100 μm.

In order to investigate, whether the detection of p75^NTR^-expressing cells is in fact associated with effective Schwann cell-mediated remyelination, we analyzed the spatial distribution of the CNS-specific myelin protein periaxin [54]. Periaxin immunoreactivity in control tissue was limited to cranial nerves (Fig 3A), and abruptly stopped once axons entered the CNS. Myelinating Schwann cells expressing periaxin were not observed in any of the lesioned areas localized in the cerebral and cerebellar grey matter, but were present in 2 (6%) and 6 (13%) lesioned areas of the cerebral and cerebellar white matter, respectively. The brain stem displayed the highest numbers of lesioned areas with periaxin-expressing Schwann cells (14 out of 38; 37%; Fig 3B). Double immunohistochemistry for periaxin and p-NF demonstrated that a large proportion of periaxin-positive Schwann cells were in fact enwrapping p-NF-positive axons (Fig 3C), substantiating factual Schwann cell remyelination. In addition, we performed double-labelling for P0 and periaxin in representative canine encephalitic lesions. Virtually all periaxin-positive Schwann cells co-expressed P0 (Fig 3D, 3E and 3F), similar to what is observed in mature myelinating Schwann cells of the PNS.

The occurrence of Schwann cell remyelination was exclusively associated with the presence of p75^NTR^-expressing cells in all anatomical localizations investigated (Fig 3G and 3H). However, p75^NTR^-expressing cells were also present in lesioned areas without Schwann cell remyelination. Prompted by this finding, we sub-grouped these areas in order to quantitatively analyze a possible association between both Schwann cell phenotypes. Lesioned areas of the cerebral and cerebellar white matter, and the brain stem were classified as follows: 1) lesioned areas without p75^NTR^-expressing cells (p75^NTR-^/PRX^-^ areas; white matter n = 73; brain stem: n = 14); 2) lesioned areas with p75^NTR^-expressing cells, but without Schwann cell remyelination (p75^NTR+^/PRX^-^ areas; white matter: n = 45; brain stem: n = 10); 3) lesioned areas with p75^NTR^-expressing cells and Schwann cell remyelination (p75^NTR+^/PRX^+^ areas; white matter: n = 8; brain stem: n = 14) and 4) normal control white matter (n = 10) and normal brain stem (n = 5) of control dogs.

**Fig 4:**
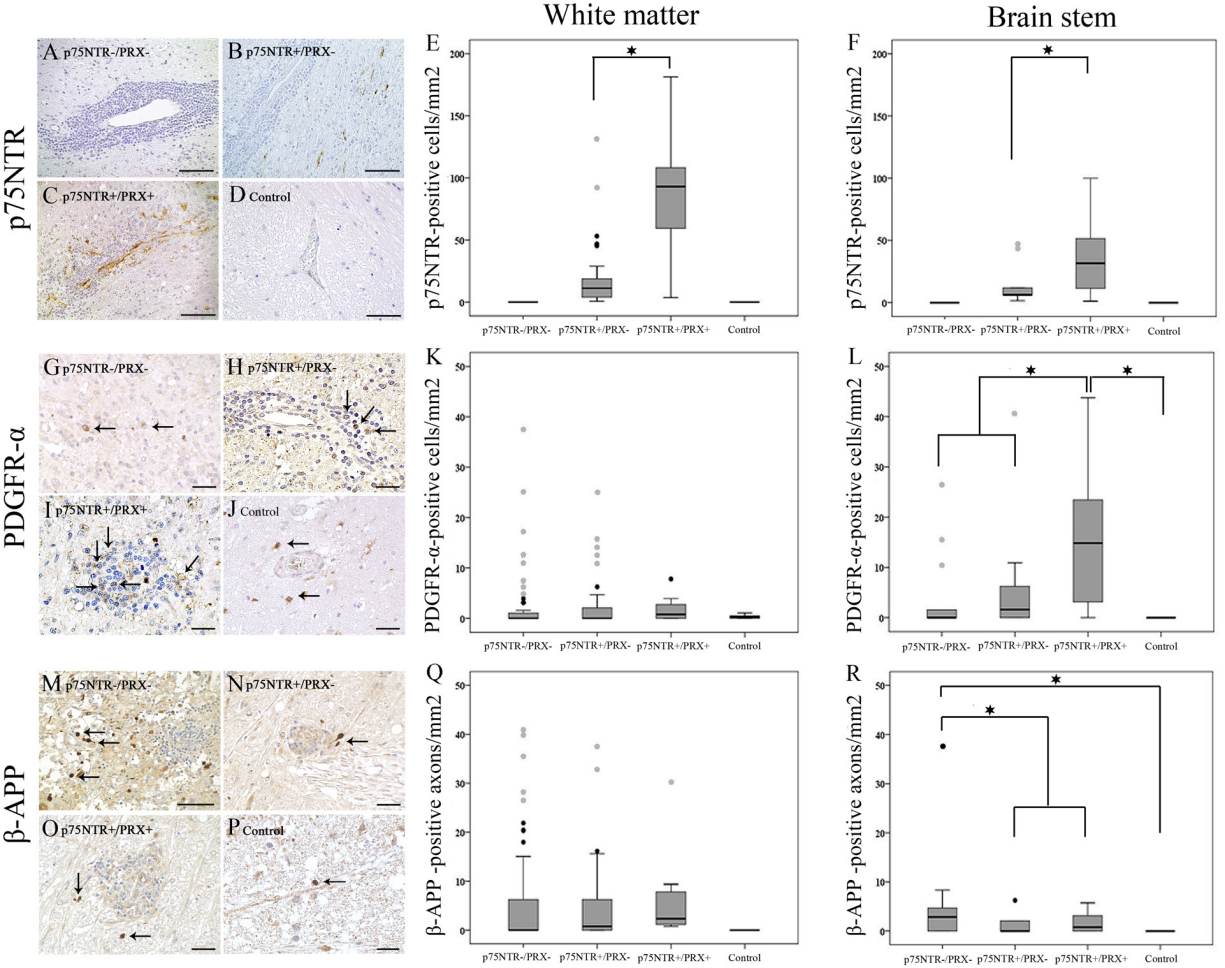
Representative immunohistochemical staining of the brain stem and box-plots of cerebral and cerebellar white matter and brain stem illustrating differences in the number of p75^NTR^-(A–F), PDGFR-α-(G–L) and β-APP-(M–R) positive cells per mm² in p75^NTR-^/PRX^-^, p75^NTR+^/PRX^-^, and p75^NTR+^/PRX^+^ areas as well as unlesioned control tissue. The number of p75 neurotrophin receptor (p75^NTR^) immunopositive cells (A, B, C, D; brain stem; brown) in the cerebral and cerebellar white matter (E) and brain stem (F) is significantly higher in p75^NTR+^/PRX^+^ areas in comparison to p75^NTR+^/PRX^-^ areas. There are no differences in the number of PDGFR-α-positive OPCs (G, H, I, J; brain stem; brown; arrows) between any groups of lesioned areas and control tissue, localized in the cerebral and cerebellar white matter (K). In the brain stem (L), p75^NTR+^/PRX^+^ areas display significantly higher numbers of PDGFR-α-positive OPCs compared to p75^NTR-^/PRX^-^, p75^NTR+^/PRX^-^ areas and control tissue. The number of β-APP-positive axons (M, N, O, P; brain stem; brown; arrows) in all lesioned areas within the cerebral and cerebellar white matter (Q) does not show significant differences between the lesioned areas and controls. In brain stem (R) p75^NTR-^/PRX^-^ areas show a significantly higher number of β-APP-positive axons compared to p75^NTR+^/PRX^-^, p75^NTR+^/PRX^+^, and control tissue. Significance was designated as p < 0.05, as detected by Mann-Whitney-U group wise test. All box-plots show median value, lower and upper quartiles, minimum and maximum (excluding outliers). Black circle: extreme value; grey circle: outlier; asterisk: significant differences between respective groups. Scale bars: (A–D) and (M) 100 μm; (G–J), (N) and (O) 50 μm; (P) 20 μm.

Within the cerebral and cerebellar white matter, and in the brain stem (Fig 4A, 4B, 4C and 4D), the number of p75^NTR^-expressing cells was significantly higher in p75^NTR+^/PRX^+^ areas in comparison to p75^NTR+^/PRX^-^ areas (p = 0.01; Fig 4E and 4F). Spearman’s rank correlation coefficient revealed a mild to moderate correlation between the numbers of cells expressing p75^NTR^ and periaxin of 0.423 and 0.759 (p < 0.01) in the cerebral and cerebellar white matter and brain stem, respectively. Interestingly, in p75^NTR+^/PRX^+^ lesioned areas, around 83% of p75^NTR^-expressing cells were found to co-localize with Sox2, while a comparatively lower amount of 42% co-expressed both markers in p75^NTR+^/PRX^-^ lesioned areas.

### Comparative quantitative analysis of p75^NTR^-expressing cells, myelinating Schwann cells, oligodendrocyte precursors cells (OPCs) and central axonal damage in canine non-suppurative meningoencephalitis

Comparative quantitative analysis of PDGFR-α as a marker for OPCs revealed that there were no significant differences between the number of PDGFR-α-positive OPCs in p75^NTR-/^PRX^-^, p75^NTR+/^PRX^-^, p75^NTR+/^PRX^+^ lesioned areas and control dogs in the cerebral and cerebellar white matter. In the brain stem (Fig 4G, 4H, 4I and 4J), however, p75^NTR+/^PRX^+^ lesioned areas displayed significantly higher numbers of PDGFR-α-positive OPCs compared to p75^NTR-/^PRX^-^, p75^NTR+^/PRX^-^, and control brain stem (Fig 4K and 4L).

β-APP expression as a marker for axonal damage [22, 37] showed that all cerebral and cerebellar white matter lesions displayed significantly higher numbers of β-APP-positive axons as compared with controls, with no significant differences between the different lesion types. However, brain stem (Fig 4M, 4N, 4O and 4P) areas lacking p75^NTR^-positive Schwann cells displayed a significantly higher number of β-APP positive axons compared to p75^NTR+^/PRX^-^, and p75^NTR+^/PRX^+^ lesioned areas (Fig 4Q and 4R).

### Comparative quantitative analysis of p75^NTR^-expressing cells, myelinating Schwann cells, astrocytes and inflammatory infiltrates

**Fig 5:**
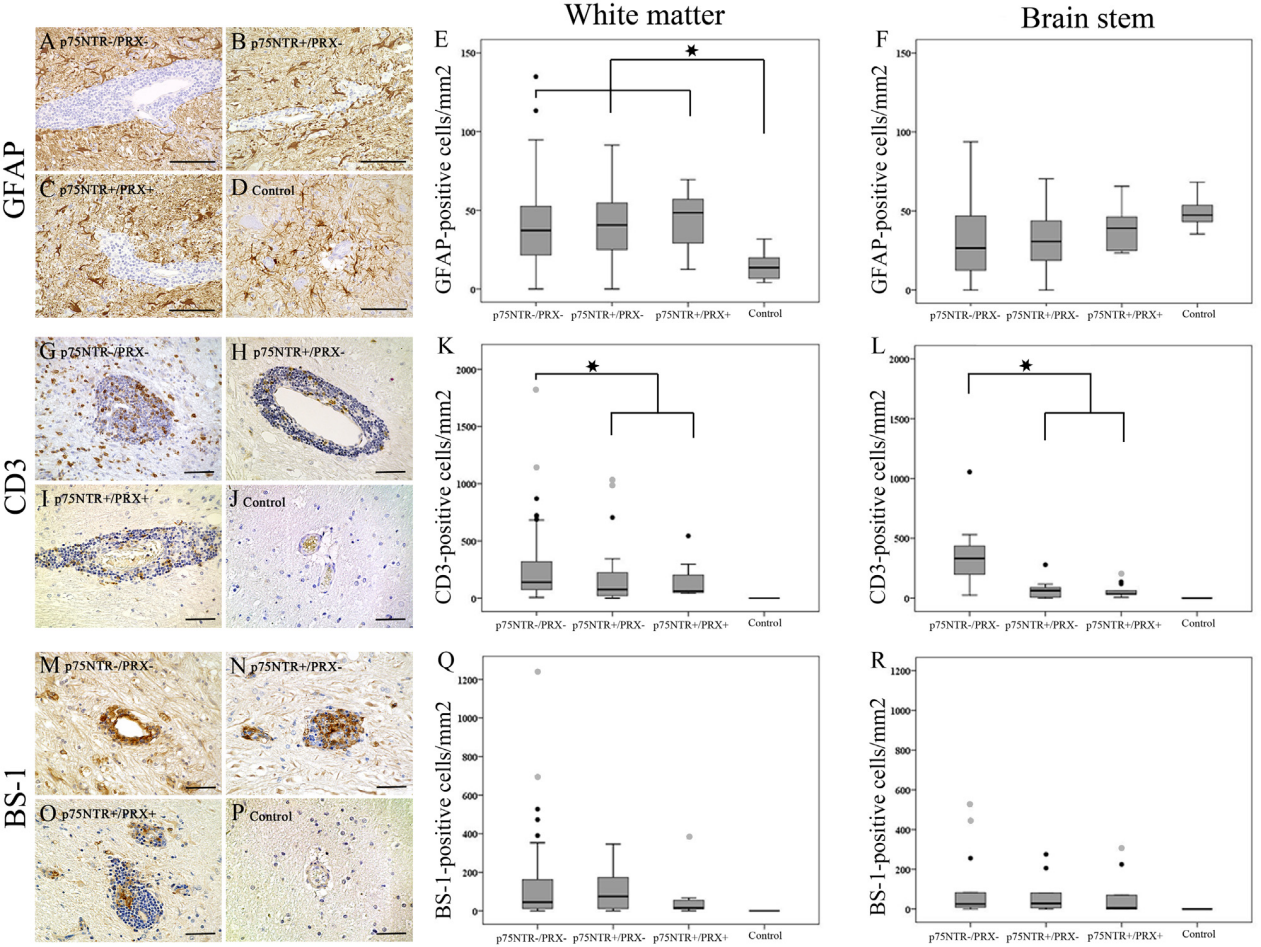
Representative immunohistochemical and lectin histochemical staining of the brain stem and box-plots of cerebral and cerebellar white matter and brain stem illustrating differences in the number of GFAP-(A–F), CD3-(G–L) and BS-1-(M–R) positive cells per mm² in p75^NTR-^/PRX^-^, p75^NTR+^/PRX^-^, and p75^NTR+^/PRX^+^ areas as well as unlesioned control tissue. GFAP-positive cells (A, B, C, D; brains stem; brown) are significantly higher in number in all lesioned groups of the white matter (E) compared to control tissue. There is no difference in GFAP immunoreactivity in the brain stem compared to controls (F). CD3-positive cells (G, H, I, J; brain stem; brown) are significantly lower in number in p75^NTR+^/PRX^+^ and p75^NTR+^/PRX^-^ lesions, when compared to p75^NTR-^/PRX^-^ areas in the cerebral and cerebellar white matter (K) and in the brain stem (L). The number of BS-1-positive microglia/macrophages (M, N, O, P; brain stem; brown) lacks significant variations between the lesioned groups in any anatomical localization (Q, R). Significance was designated as p < 0.05, as detected by Mann-Whitney-U group wise test. All box-plots show median value, lower and upper quartiles, minimum and maximum (excluding outliers). Black circle: extreme value; grey circle: outlier; asterisk: significant differences between respective groups. Scale bars: (A–D) 100 μm; (G–J) and (M–P) 50 μm.

Large hypertrophic astrocytes were present in all lesioned areas. GFAP immunoreactivity revealed a marked astrogliosis in p75^NTR-^/PRX^-^, p75^NTR+^/PRX^-^, and p75^NTR+^/PRX^+^ lesioned areas localized in the cerebral and cerebellar white matter as compared with controls. However, there was no difference in its expression between the three lesion types. In the brain stem (Fig 5A, 5B, 5C and 5D), GFAP-expression did neither significantly vary between the investigated lesion types nor between lesions and controls (Fig 5E and 5F).

In the cerebral and cerebellar white matter and in the brain stem, CD3-positive T lymphocyte (Fig 5G, 5H, 5I and 5J) numbers were significantly lower in p75^NTR+^/PRX^+^ and p75^NTR+^/PRX^-^ lesions when compared to p75^NTR-^/PRX^-^ areas (Fig 5K and 5L). The number of BS-1-positive microglia/macrophages (Fig 5M, 5N, 5O and 5P) did not show any significant differences between the lesion groups concerning the anatomic localization (Fig 5Q and 5R). CD3-positive T lymphocytes and BS-1-positive microglia/macrophages were not observed in any anatomical localization of control dogs.

### Appearance of p75^NTR^/Sox2 expressing cells during culturing of organotypic slices from the normal adult canine brain stem

**Fig 6:**
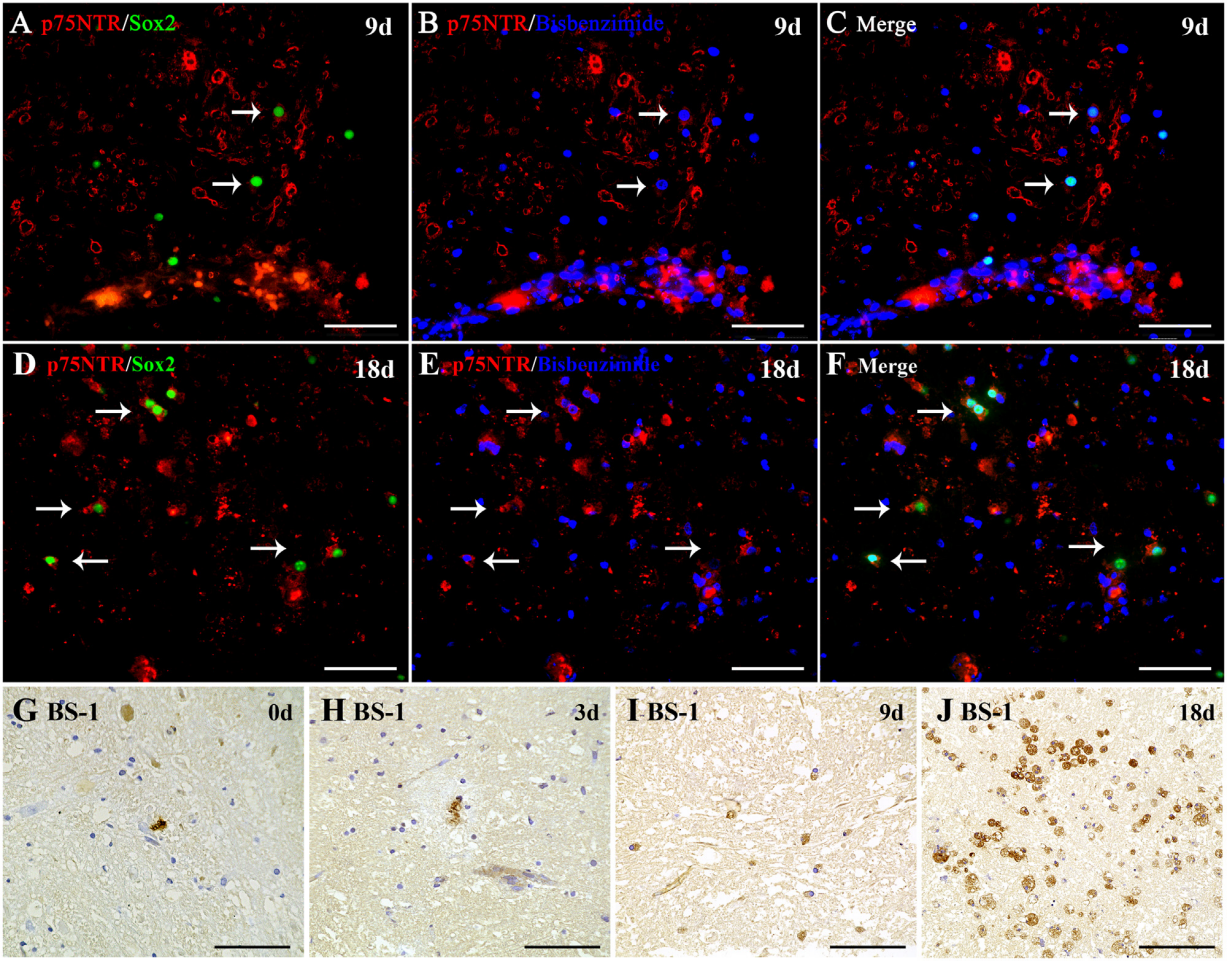
Double immunofluorescence staining of p75^NTR^ (red) and Sox2 (green), p75^NTR^ and nuclear counterstaining with bisbenzimide (blue), and merged pictures of p75^NTR^, Sox2, bisbenzimide at day 9 of cultivation (A,B,C) and day 18 of cultivation (D,E,F) in organotypic slice cultures from the normal adult canine brain stem. During culturing, relatively small numbers of p75^NTR^ (red) and Sox2 (green, nuclear staining) co-expressing cells (arrows) are detected in the brain parenchyma in close proximity to blood vessels by day 9 (A,B,C), but increase in number by day 18 (D,E,F) of culturing. (A-F) scale bars: 20 μm. BS-1 lectin histochemistry (G-J) demonstrates the lack of microglial and macrophage-like cells at the beginning of the cultivation period (G; 0 days) and a massive increase in the number of BS1-positive macrophages/microglia during cultivation (H, 3 days; I, 9 days), reaching their highest numbers by day 18 of culturing (J). (G-J) scale bars: 50 μm.

To gain further insights into the possible origin of Schwann cells, slice cultures from the brain stem of four normal control dogs were established and examined at different time points. The experiments focused on the co-expression of p75^NTR^ and Sox2, which appeared within Virchow-Robin vascular spaces, and in close proximity to blood vessels and cranial nerve entry *in situ*. In addition, mature myelinating Schwann cells were identified by the expression of periaxin. Co-labeling of p75^NTR^ and Sox2 in all four dogs revealed the emergence of bi- to multipolar cells 9 days after slicing (Fig 6A, 6B and 6C) increasing considerably in number at day 18 (Fig 6D, 6E and 6F). Strikingly, p75^NTR^-Sox2 co-expressing cells were mainly localized in close proximity to blood vessels at both time points. Until day 18 *in vitro*, no periaxin-positive cells, indicative of mature, myelinating cells, were detected. Lectin histochemistry using BS-1 demonstrated the lack of microglial and macrophage-like cells at the beginning of the cultivation period and a massive increase in the number of BS1-positive cells during culturing (Fig 6G, 6H, 6I and 6J). These cells were of round to oval morphology and displayed a vacuolated cytoplasm reminiscent of actively phagocytizing macrophages.

## Discussion

Despite rapid advances in understanding the cellular and molecular events triggering remyelinating Schwann cells following CNS injury, relatively little is known about Schwann cell origin and factors contributing to the occurrence of these cells in spontaneous diseases such as MS and other inflammatory CNS conditions [8, 9, 10, 11, 12, 21]. In an attempt to address these open points, the present investigation focused on the spatial distribution and phenotype of p75^NTR^-expressing cells and remyelinating Schwann cells at the lesion site in naturally occurring, canine idiopathic CNS inflammatory conditions. p75^NTR^ has been widely used as a prototype marker for Schwann cells. However, p75^NTR^ alone is not sufficient for their unequivocal identification in the CNS, since previous in situ studies revealed that other cell types such as OPCs and astrocytes are similarly able to express p75^NTR^ [3, 5, 47, 56, 57, 60, 61]. In addition, a spectrum of unique CNS glia, collectively introduced under the term aldynoglia share the expression of p75^NTR^ [62, 63, 64, 65]. In fact, Schwann-like brain glia may represent a premyelinating state generated from progenitors of the oligodendrocytic lineage [22, 23, 24]. If this is the case, one would expect co-expression of both PDGFR-α and p75^NTR^ in OPCs, which once differentiated, lose PDGFR-α expression but retain p75^NTR^ [22].

One major drawback in studying such a heterogeneous population with naturally occurring diseases, different genetic backgrounds, different ages and sexes is represented by the fact that it cannot be completely ruled out that individual backgrounds and other, so far undetermined factors, may have influenced the results, *i*.*e*. the occurrence of p75^NTR^-expressing cells, though the anamnestical data in table 1 suggest that neither age, sex, and lesion severity fully explain the interindividual variability. Thus, larger study cohorts are needed to fully elucidate possible individual factors, which explain why some subjects exhibit occurrence of p75^NTR^-expressing cells, while others do not.

The identity of p75^NTR^-expressing cells was further elucidated by detailed double immunofluorescence staining demonstrating that in this natural occurring CNS inflammatory condition, Schwann cells recapitulated differentiation patterns described after peripheral nerve injury [66, 67, 68]. Axonal damage in the PNS induces dedifferentiation of mature Schwann cell [47, 67], a process requiring down-regulation of myelin genes, such as P0, periaxin and Egr-2/Krox20, and re-expression of molecular markers including p75^NTR^, GFAP and the transcription factor Sox2 [47, 51]. In the present case, p75^NTR^-expressing cells within the injured CNS co-labeled with Sox2, but not with GFAP and GAP-43, two markers described in immature Schwann cells and in the mature non-myelinating form [5, 47, 48], as confirmed in sciatic nerves of controls by co-labeling with p75^NTR^. In addition, these cells did not stain with antibodies against Egr2/Krox20 and periaxin suggesting a down-regulation of myelin markers to gain a dedifferentiated phenotype [47, 51]. A similar up-regulation of p75^NTR^ and Sox2, and lack of co-expression of p75^NTR^ with GFAP, GAP-43, Egr2/Krox20 and periaxin was observed in Schwann cells in the sciatic nerve of a dog suffering from degenerative neuropathy, thus substantiating that p75^NTR^/Sox2-positive cells within the injured CNS might in fact represent dedifferentiated Schwann cells. Although GFAP has been demonstrated to be also up-regulated by dedifferentiated Schwann cells after axotomy in rodent models [47, 67], it remains unclear whether the lack of its expression in the present investigation in both, injured sciatic nerve and CNS might simply reflect species specific differences. However, the authors admit that the low *n* of peripheral nerves studied does not permits definitive conclusions. Thus, further investigations on both diseased and healthy canine peripheral nerves are needed in order to obtain more information on the different phenotypes of Schwann cells in canine peripheral nerves.

It is not until axons are completely regenerated that dedifferentiated Schwann cells regain their myelinating phenotype and initiate remyelination of PNS axons [6, 66]. The number of p75^NTR^-expressing immature/dedifferentiated Schwann cells has been shown to decline in parallel to the appearance of the myelinating cells, postulating a redifferentiation of p75^NTR^-expressing Schwann cells within in the injured spinal cord [21]. However, we found that p75^NTR^/Sox2-expressing cells were higher in number in lesioned areas containing mature myelinating Schwann cells. It is well known that Sox2 is an inducer of cell proliferation after PNS injury [69, 70, 71]. Similar to the involvement of p75^NTR^ in Schwann cell migration [72], Sox2 has been shown to be involved in early stages of peripheral nerve repair. For instance, Sox2 orchestrates ephrin-B/EphB2-mediated Schwann cell sorting followed by directional cell migration to guide regrowing axons [50]. We therefore speculate that the emergence of p75^NTR^/Sox2 expressing cells within the injured CNS may represent an early step for an effective CNS regenerative process. Whether the same environmental cues as in the PNS acts as an attractant for p75^NTR^/Sox2 expressing cells in the injured CNS remains to be determined.

Although the model in this study does not allow the characterization of chronological changes regarding the phenotype of Schwann cells, both immunohistochemical data and the particular distribution of Schwann cells around blood vessels and the proximity to cranial nerve entries suggest that remyelinating Schwann cells might be derived from p75^NTR^/Sox2-expressing Schwann cells that invaded the CNS from the periphery. Similar results were found in a study using linage-tracing of Schwann cells after spinal cord injury [21] and in naturally occurring spinal cord trauma [19]. Under both conditions, mature myelinating Schwann cells of spinal nerve roots dedifferentiated into p75^NTR^-expressing Schwann cells followed by migration into the CNS to regain their myelinating phenotype. On the other hand, the detected myelinating Schwann cells in this study characteristically co-expressed P0 and periaxin, two myelin markers observed in PNS-derived remyelinating Schwann cells [11]. Thus, taken together, these data provide evidence that PNS-derived Schwann cells highly contribute to the Schwann cell population in naturally occurring CNS inflammatory conditions. In this respect, both cranial nerves and autonomic nerves of blood vessels can be considered as potential sources, extending previous observations [12, 20, 73, 74]. Strikingly, we provide further evidences that non-myelinating Schwann cells of autonomic nerves regain a more dedifferentiated phenotype well before invading the CNS parenchyma, since p75^NTR^/Sox2 expressing cells lacking GFAP, another marker for immature and mature non-myelinating Schwann cells in the PNS [48] were detected directly inside Virchow-Robin perivascular spaces in all investigated localizations, and they emerged *in vitro* in organotypic slices in close proximity to blood vessels. However, PDGFR-α-expressing cells were present within lesioned areas with and without p75^NTR^-expressing cells and remyelinating Schwann cells. Thus, the presence of OPCs might point to the fact that indeed some of the detected p75^NTR^ cells might derive from OPCs, though we did not detect any co-labelling of PDGF-α and p75^NTR^. Thus, in the absence of tracing studies we are unable to definitely conclude on the exact origin of the detected cells.

It is well known that dedifferentiated Schwann cells provide a supportive environment for axonal regeneration after PNS injury before remyelination starts [7, 50, 75]. However, there is apparently a lack of consensus regarding functional consequences of such cells within the injured CNS [10, 11, 19, 21, 76]. In this study, axonal damage in the brain stem, as detected by axonal immunoreactivity for β-APP, was found to be significantly lower in lesioned brain stem areas with different stages of Schwann cells as compared to lesions without such cells. However, as reduced axonal damage does not necessarily imply enhanced axonal regeneration, further studies with markers for axonal regeneration are essential to substantiate these findings.

It has generally been assumed that the absence of astrocytes is critical for Schwann cell migration into the injured spinal cord, both under experimental and natural conditions [20, 77, 78, 79, 80]. Strikingly, the present study on naturally lesioned brain tissue demonstrated that the occurrence of Schwann cells within the cerebral and cerebellar white matter and the brain stem was not dependent on the presence of astrocytes. Thus, these data provide novel *in situ* evidence that Schwann cells with an immature phenotype might be less susceptible to astrocytic presence. In fact, previous experiments demonstrated that immature Schwann cells grafted to the lesioned CNS have a greater ability to migrate and intermingle with astrocytes than mature Schwann cells [19, 75, 81, 82]. Moreover, in a recent study using organotypic brain slices of adult mice, p75^NTR^-positive bi-polar cells were similarly observed independent of GFAP expression [46].

More recently, macrophages have been shown to play critical roles in regenerative processes following both peripheral nerve injury [83, 84] and CNS demyelinating diseases [85, 86, 87]. Although the involvement of microglia/macrophages has been mainly studied in the context of differentiation of OPCs into oligodendrocytes [85], less is known about their role in Schwann cell-mediated repair. Taking advantage of the inflammatory spontaneous canine disease used in this study, we demonstrated that there were no significant differences in the number of microglia/macrophages in lesioned areas with and without p75^NTR^-expressing Schwann cells, and with remyelinating Schwann cells. These data suggest that an increase of microglia-macrophages might not be determinant for the emergence of p75^NTR^-expressing Schwann cells or their further redifferentiation into their myelinating phenotype *in vivo*. This observation contradicts previous findings in canine and murine organotypic slice cultures [22, 46], where p75^NTR^-expressing cells have shown to increase in parallel to the occurrence of microglia/macrophages. It seems, however, that the *in situ* presence of microglia/macrophages at least provides an adequate environment for the plasticity of Schwann cells within the CNS, since we were not able to detect any Schwann cells in canine inflammatory CNS lesions dominated by neutrophils (data not shown). Such observations in part resemble, what occurs after peripheral nerve injury. Here, Schwann cell dedifferentiation starts before considerable numbers of macrophages are recruited to the lesion site [83, 84]. Both, dedifferentiated Schwann cells and macrophages are implicated in phagocytizing myelin debris within the PNS [7, 47, 88]. Whether dedifferentiated Schwann cells similarly contribute to myelin clearance within the CNS remains to be determined. Although the participation of lymphocytes during PNS regeneration has been poorly investigated [47], the *in situ* occurrence of dedifferentiated Schwann cells appears to be linked to areas with reduced numbers of T lymphocytes, possibly implying a negative influence of T cells in Schwann cell-mediated CNS repair. However, this observation remains to be substantiated by further studies.

Taken together, the present study shows that Schwann cell plasticity after CNS inflammatory conditions resembles in several aspects their response after peripheral nerve injury. In addition, it seems that dedifferentiation, invasion into CNS lesion sites, and redifferentiation of Schwann cells represent natural regenerative mechanisms not only observed in the injured spinal cord [19, 21] but also extending to the cerebral and cerebellar white matter, and the brain stem in naturally occurring CNS inflammatory conditions. Considering the potential regenerative properties of Schwann cells, the targeting of p75^NTR^/Sox2-expressing cells and the development of strategies designed to enhance their differentiation into competent remyelinating cells appears to be a promising therapeutic approach to achieve regeneration in inflammatory/demyelinating CNS diseases.
